# Blisters Don't Lie: Celiac Disease in the Skin

**DOI:** 10.1002/ueg2.70284

**Published:** 2026-08-27

**Authors:** Jonas F. Ludvigsson, Philip Curman

**Affiliations:** ^1^ Department of Medical Epidemiology and Biostatistics Karolinska Institutet Stockholm Sweden; ^2^ Department of Pediatrics Örebro University Hospital Örebro Sweden; ^3^ Celiac Disease Center College of Physicians and Surgeons Columbia University Irving Medical Center New York New York USA; ^4^ Department of Medicine (Solna) Division of Dermatology and Venereology Karolinska Institutet Stockholm Sweden; ^5^ Dermato‐Venereology Clinic Karolinska University Hospital Stockholm Sweden; ^6^ Lübeck Institute of Experimental Dermatology University of Lübeck Lübeck Germany

Dermatitis Herpetiformis is an odd occurrence. On the one hand, many patients, physicians and researchers regard it as a form of celiac disease (CeD) [1], while others see it as an independent condition only belonging to that same family of gluten‐related conditions. It is a bit like the main characters in the movie ‘Fight Club’ (1999) where what appears to be two separate individuals (the narrator and Tyler Durden) are actually the same (https://www.imdb.com/title/tt0137523/).

But what is clear, irrespective of the true relationship between DH and CeD, is that DH is burdensome, usually appears with blistering rashes, and often goes undetected for years [2]. As Ocagli and colleagues point out: ‘*its epidemiology remains poorly defined*’ [3]. This was the rationale why six Paduan researchers set out to examine the prevalence of DH among individuals with CeD [3], and they did so with flying colours!

Readers of this journal will know that CeD is a common disease (1%–2% of the general population affected), and most studies on prevalence will focus on how often *CeD* is seen in other conditions [4]. Now Ocagli and colleagues turn it around, and start with the CeD patients.

Exploring four medical databases the researchers identified more than 7000 studies, 24 of which were deemed relevant for their purpose. Based on 73,905 patients with CeD, they estimated the pooled DH prevalence to 6.8%. Somewhat surprising, the prevalence was similar (6.7%) also when restricting the study sample to non‐selected populations. It was however lower in children than in adults (the latter potentially due to DH developing after long‐term gluten). Excluding the largest study so far (Lebwohl et al., with more than 49,000 CeD patients) [5] did not impact on the overall prevalence, but when excluding all studies based on registry or administrative data, the pooled prevalence rose (8.8%), and from clinical experience we believe this figure comes closer to the true occurrence of DH in patients with CeD. However, as the authors also recognize, most of the non‐registry cohorts came from secondary or referral care, which may overrepresent symptomatic CeD and inflate this estimate; the true figure probably lies somewhere between the two.

However, the Italian team also noted a very high heterogeneity (98.9%) indicating large between‐study differences, suggesting that standardised diagnostic approaches when examining CeD and investigating patients for DH would be beneficial. We agree.

The diagnostic gold standard remains direct immunofluorescence of perilesional skin, demonstrating granular IgA deposits in the dermal papillae [6]. Yet this is often not the first test performed, and because the eruption is polymorphic and intensely itchy, DH is readily mistaken for other conditions such as atopic dermatitis, eczema, prurigo, urticaria, scabies, or other autoimmune blistering diseases such as bullous pemphigoid and linear IgA bullous dermatosis [7].

The authors are to be commended for their meta‐analysis. They follow the PRISMA guidelines, they cover several databases, and they go through more than 7000 records. This is not an easy task, and it does not come as a surprise that while they started their literature reviews in July 2023, they felt they had to update their search one and a half year later (February 2025), most likely since a literature review takes time. As for most meta‐analyses about CeD, the majority of studies in the current meta‐analysis [3] were European, but as the interest in and awareness of CeD is picking up, the number of studies outside Europe has increased and the Ocagli study included papers also from North America, Asia and South America.

The authors do a good job outlining the limitations of their study, but no shortcoming should eclipse the value of this paper. In this paper about DH, Ocagli and colleagues highlight an important phenotype and clinical expression of CeD, that can both serve as a signal of undiagnosed CeD but also merit treatment of its own [8, 9].

Finally, we once more take off our hats for the authors' literature search. So as not to miss a single publication about DH, they even searched for the synonym “Duhring disease”. As noted, Louis Adolphus Duhring (1845–1913) [10] first described DH in 1894. He went on to write another 17 papers about the disease until its existence was accepted. Duhring was one of the founders of the American Dermatological Society.

We believe he would have liked the paper by Ocagli and colleagues very much.

## Conflicts of Interest

Dr Ludvigsson has received financial support from Merck/MSD for a study on inflammatory bowel disease and fibrosis; and for developing a paper reviewing national healthcare registers in China. Dr Ludvigsson also has an ongoing research collaboration on celiac disease and on chronic liver disease with Takeda. Earlier support includes a grant from Janssen for an unrelated study on behalf of the Swedish IBD quality register (SWIBREG). Dr Curman has no relevant conflict of interest to declare.

## Data Availability

Data sharing not applicable to this article as no datasets were generated or analysed during the current study.
